# Genomic-Destabilization-Associated Mutagenesis and Clonal Evolution of Cells with Mutations in Tumor-Suppressor Genes

**DOI:** 10.3390/cancers11111643

**Published:** 2019-10-24

**Authors:** Ken-ichi Yoshioka, Yusuke Matsuno, Mai Hyodo, Haruka Fujimori

**Affiliations:** 1 Division of Carcinogenesis and Cancer Prevention, National Cancer Center Research Institute, 5-1-1 Tsukiji, Chuo-ku, Tokyo 104-0045, Japan; yumatsun@ncc.go.jp (Y.M.); mhyodo@ncc.go.jp (M.H.); harfujim@ncc.go.jp (H.F.); 2 Department of Applied Chemistry, Faculty of Science, Tokyo University of Science, 1-3 Kagurazaka, Shinjuku-ku, Tokyo 162-8601, Japan; 3 Biological Science and Technology, Tokyo University of Science, Niijuku, Katsushika-ku, Tokyo 125-8585, Japan

**Keywords:** clonal evolution, genomic instability, cancer development

## Abstract

The development of cancer is driven by genomic instability and mutations. In general, cancer develops via multiple steps. Each step involves the clonal evolution of cells with abrogated defense systems, such as cells with mutations in cancer-suppressor genes. However, it remains unclear how cellular defense systems are abrogated and the associated clonal evolution is triggered and propagated. In this manuscript, we review current knowledge regarding mutagenesis associated with genomic destabilization and its relationship with the clonal evolution of cells over the course of cancer development, focusing especially on mechanistic aspects.

## 1. Introduction

Cancers generally develop as a consequence of mutations, which can be divided into two types: passenger or driver mutations. Unlike the former, the latter is much less frequent and is critical for the clonal evolution of cells progressing through the different stages of cancer development. Driver mutations include mutations that cause dysfunction of cancer-suppressor genes and those that enhance cancer gene function. It is still unclear how those mutations are induced and whether they and their associated cancers can be prevented. Indeed, these issues are a longstanding subject of controversy. A standard view is that cancer mutations can be categorized into three types: hereditary, replicative, and environmental; the environmental mutations are avoidable, whereas the others are mostly unavoidable [1]. The majority of mutations are replicative, arising randomly during DNA replication, implying that most cancer-driver mutations and associated cancers are unavoidable [1,2,3]. Accordingly, secondary prevention is a major priority in efforts to prevent death from cancer.

Mutations accumulate widely, even in normal cells, over the course of aging (e.g., in the human esophagus [4,5] and skin [6]). Surprisingly, these mutations include mis-sense and non-sense mutations in cancer-driver genes at positions identical to those in cancer cells. These observations suggest that cancer development is not directly initiated by these mutations, raising the question of how cancer is initiated.

Using an in vitro model, a more recent study reported that mutations occur frequently in association with genomic destabilization when cells experience replication stress [7]. These genomic-destabilization-associated mutations are further associated with the clonal expansion of cells mutated in cancer-driver genes [7]. In breast cancer cells, it is well established that genomic destabilization occurs in association with the accumulation of replication-stress-associated double strand breaks (DSBs) that are often caused by oncogene acceleration [8]. Given that most cancers ultimately develop genomic instability [9,10], the mutations associated with genome instability must occur widely in many types of cancer cells.

In this review, we focus mainly on genomic instability induced in the nucleus, its association with mutations, and the resultant clonal evolution of cells with abrogated defense systems, which has been recently clarified [7]. Mutations are induced when replication-stress-associated DSBs accumulate and are clearly different from replicative mutations that occur randomly as the result of errors introduced during canonical replication. Importantly, the mutations associated with genomic destabilization may be tightly associated with the clonal expansion of cells mutated in cancer-driver genes.

## 2. Mutations Induced along with Genomic Destabilization

### 2.1. Mutation Rate and Its Association with Cancer Risk

In normal human cells, DNA is usually replicated by high-fidelity polymerases δ and ε [11,12,13]. Errors caused by those polymerases are primarily repaired by their proofreading system (Table 1), and the remaining errors are targeted by the mismatch repair (MMR) system [14,15,16]. 

Consequently, mutation is largely suppressed during normal replication. Deficiencies in MMR and proofreading performed by polymerase ε are associated with hypermutation and cancer predisposition [16,26,27]; thus, the mutation rate is strongly associated with cancer risk. However, this association between mutation rate and cancer risk may be unrelated to cell division frequency and resultant changes in cell number, as aptly pointed out by Peto’s paradox: cancer incidence does not appear to correlate with the number of cells in an organism at the species level [28].

Mutations in cancer cells are not induced randomly. Based on whole-genome and exome analyses, chromatin loci with higher mutation levels can be characterized as (I) gene loci that are not highly expressed [29], (II) chromosome regions replicating in late S phase [29], (III) heterochromatic loci [30], and (IV) loci with closed chromatin that is not sensitive to DNase I [29,30,31]. Mutation rates differ by severalfold between high- and low-mutation loci. Although the source of these differences remains unclear, the phenomenon might be associated with genomic instability, as heterochromatic loci are often subjected to replication stress and chromosomal rearrangements [29,32,33]. Consistent with this, a recent study reported that hypermutation is associated with genomic destabilization [7].

### 2.2. Mutation Induction when Replication-Stress-Associated DSBs Accumulate

**Figure 1:**
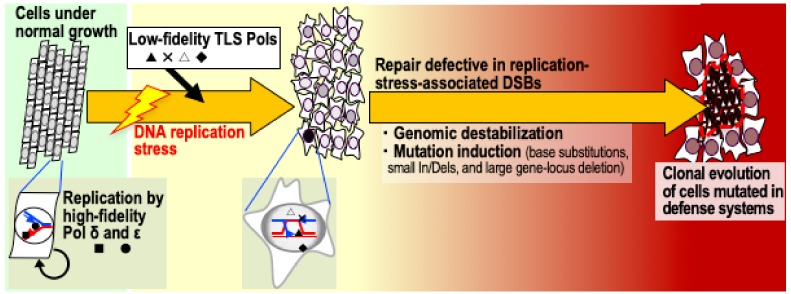
Model of replication-stress-triggered induction of clonal evolution through genomic destabilization. Cells that accumulate replication-stress-associated double strand breaks (DSBs) are at higher risk of genomic destabilization of either chromosomal instability (CIN) or microsatellite instability (MSI). Genomic destabilization is associated with mutation in cancer-driver genes, leading to the clonal evolution of cells with defects in cellular defense systems.

Among the many mutations induced in cancer cells, somatically-induced replicative mutations are the most prominent [1]. Based on a recent study, such replicative mutations can be separated into two types: those induced as random errors during canonical replication, and those induced in association with replication-stress-associated DSBs that accumulate in association with genomic destabilization [7]. Unexpectedly, the former type is relatively rare even in MMR-deficient cells that cannot correct errors during replication [7]. By contrast, in the latter, hypermutation occurs in association with DSBs and genomic destabilization in barely replicating cells (Figure 1).

Importantly, cancer-driver mutations arise during the latter; the induced mutations include base substitutions, small insertions and deletions, and large deletions of entire genes [7] (Figure 1). The mechanisms responsible for mutations in this context are not clear, probably because of the presence of repair systems and other related polymerases (Table 1). Indeed, these DSBs are generally a target of homologous recombination (HR), which does not involve high-fidelity polymerases (pols) δ and ε. Instead, low-fidelity translesion synthesis (TLS) polymerases δ, κ, and θ, which synthesize DNA over lesioned templates [20,21,34], are highly expressed [7,17,18,19]. Thus, the higher mutation rates are probably due to DNA synthesis performed by these polymerases, which lack proofreading activity, and the errors probably occur during translesion synthesis. Thus, while mutations are strongly suppressed during normal cell division, they increase after replication-stress-associated DSB accumulation and genomic destabilization. Given that most cancers ultimately develop genomic instability [9,10], these mutations are most likely the ones that contribute to cancer progression [7].

### 2.3. Senescence-Associated Increase in the Risk of Genomic Destabilization

**Figure 2:**
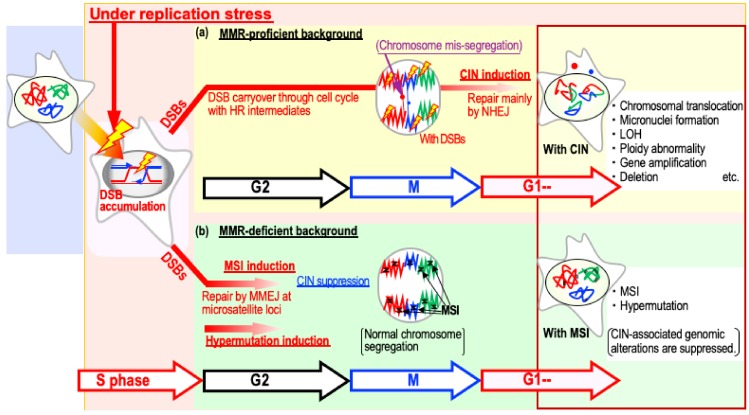
Model of distinct chromosomal instability (CIN) and microsatellite instability (MSI) induction processes triggered by replication stress. Induction of both CIN and MSI is triggered by replication stress. Although CIN arises when replication-stress-associated double strand breaks (DSBs) are not effectively repaired by homologous recombination (HR), MSI arises when those DSBs are erroneously repaired by microhomology-mediated end joining (MMEJ) in an mismatch repair (MMR)-deficient background. MSI induction is associated with the induction of hypermutation—a context in which CIN induction is generally suppressed. NHEJ: non-homologous end joining.

Most cancer cells develop one of two forms of genomic instability: chromosomal instability (CIN) or microsatellite instability (MSI) [9]. CIN is the most frequent, but MSI usually arises in MMR-deficient cancers (Table 1). The induction of both CIN and MSI is triggered by replication-stress-associated DSBs [7,22,35,36] (Figure 2). 

**Figure 3:**
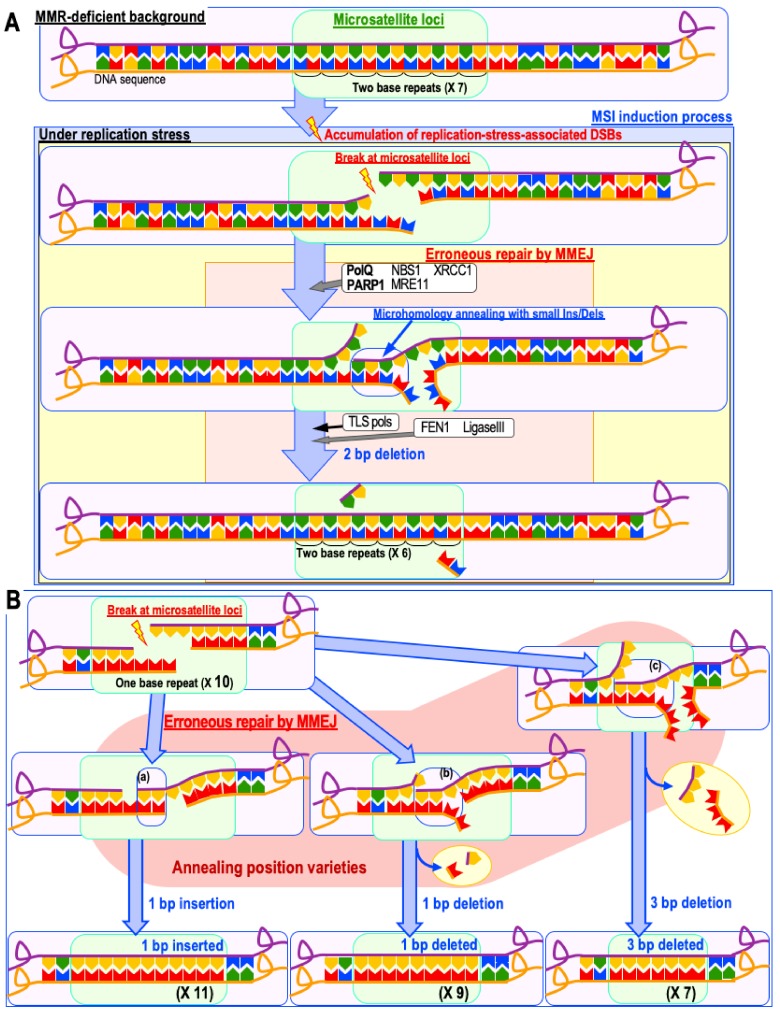
Model of microsatellite instability (MSI) induction. (**A**,**B**) Under an mismatch repair (MMR)-deficient background, double strand breaks (DSBs) caused by replication stress are effectively repaired by microhomology-mediated end joining (MMEJ), which is mediated by PolQ and Poly (ADP-ribose) polymerase 1 (PARP1) (**A**). Such microsatellite loci could be repeats of a single base (**B**), two bases (**A**), or more. This induces insertions and deletions of a few bases, specifically at repetitive sequence loci (i.e., microsatellite loci), leading to MSI induction. Because microhomologies can anneal in multiple ways (a–c), this process could lead to multiple types of insertions and deletions (**B**).

CIN often occurs when such DSBs are not effectively repaired by HR, that is, when DSBs arising during replication are carried over into the following G1 phase at a time when HR is not functional. Consequently, the DSBs are often erroneously ligated by non-homologous end joining (NHEJ) with topologically incorrect broken ends, resulting in a background that causes CIN-associated chromosomal alterations such as chromosomal translocations, deletions, and rearrangements [37]. By contrast, MSI arises when those DSBs are effectively but erroneously repaired by microhomology-mediated end joining (MMEJ; also known as alternative end joining) [7] (Figure 3), during which CIN is suppressed because of the resultant DSB repair (Figure 2). Thus, both CIN and MSI are triggered by replication-stress-associated DSBs and arise due to abnormalities in the repair of these lesions.

Most cancers develop genomic instability due to abnormalities in DSB repair; paradoxically, however, most lack background mutations in DNA repair systems [38,39]. This raises the question of how genomic destabilization occurs even in normal cells, that is, genetically normal cells with functional DNA repair systems. This phenomenon is probably associated with age, which is associated with an elevated cancer risk [40,41]. Indeed, irreparable DSBs accumulate widely in association with age in vivo and passage in vitro [42]. Consistent with this, cells often undergo senescence as a consequence of DSB accumulation [42,43,44,45,46,47]. Although the reasons for DSB accumulation are not clear, they are likely to include a reduction in histone H2AX, which mediates repair-factor recruitment and is required for genome stability [48]. 

Indeed, the growth arrest of normal cells is associated with reduced levels of H2AX after serial proliferation [49,50,51]. Cells in the H2AX-diminished state are vulnerable to exogenous growth acceleration, causing them to accumulate DSBs in association with replication stress and subsequently develop genomic instability [49]. Repair deficiency and the associated risk of genomic destabilization may be specific to the DSBs caused by replication stress, as cells in this state barely repair replication-stress-associated DSBs; however, they can still effectively repair DSBs caused by irradiation through transient stabilization of H2AX under the control of ATM and SIRT6 [52,53].

### 2.4. Genomic-Destabilization-Associated Mutagenesis in Cancer Development

Based on the aforementioned Mouse Embryonic Fibroblasts (MEF) model, it is clear that genomic destabilization is associated with mutagenesis and the resultant clonal evolution of cells with mutations in cancer-driver genes (e.g., the ARF/p53 module) [7]. An important question is whether such genomic-destabilization-associated mutagenesis is truly relevant to the mutations induced in cancer cells. Currently, the best support for this is the higher mutation rates at heterochromatin loci [29], which are also hotspots of genomic destabilization [32,33]. Thus, loci with higher mutation rates are tightly associated with loci identified as hotspots of genomic destabilization.

Kataegis, localized hypermutations at genomically destabilized loci, is another cause of genomic-destabilization-associated mutagenesis [54,55,56]. This type of mutation includes C > T transitions, mediated by the deaminase APOBEC3A/B, at CpG loci and others [55,56,57,58]. Mutations caused by reactions other than deamination are probably due to errors during DNA synthesis, likely in association with background expression of low-fidelity TLS polymerases when cells accumulate replication-stress-associated DSBs [7]. These observations suggest that DNA loci subjected to repair of replication-stress-associated DSBs are widely synthesized by low-fidelity TLS polymerases, which lack a proofreading function.

### 2.5. Epigenetic Alterations as a Cause of Genomic Destabilization

The risk of genomic destabilization [8] might be increased by epigenetic alterations, including epigenetic silencing of DNA repair and damage response factors such as MLH1, ATM, CHK2, MGMT, Ogg1, MBD4, and NEIL1. The most prominent pathway is the epigenetic silencing of the Mlh1 gene, resulting in MMR deficiency [59]. In fact, besides its involvement in cancers associated with Lynch syndrome, MSI is observed in many sporadic cancers, which represent about 15%–20% of colorectal and ovarian cancers [60,61]. Most of these cancers show MMR deficiency as a result of epigenetic silencing of the Mlh1 gene [59,60,61]. In these cancers, the primary risk factor for the development of cancer is epigenetic alterations.

### 2.6. Avoidable or Unavoidable?

An important question is whether mutations that promote cancer development are avoidable. Based on current knowledge, the critical question now is whether mutations caused by replication stress in association with genomic destabilization are avoidable, as they are tightly associated with the clonal evolution of cells with mutations in cancer-driver genes [5,6]. Unlike errors that are randomly induced during canonical replication, mutations caused under replication stress are theoretically avoidable by mechanisms devoted to maintaining genome stability. In fact, as shown in the MEF model, immortalization dependent on mutations in the ARF/p53 module is blocked when genome stability is maintained by cultivating cells with reduced levels of replication stress [50]. Thus, cancers that develop due to genomic instability might be avoidable through maintenance of genome stability. However, it remains unclear how genome stability could be maintained. Therefore, to truly prevent cancers using this strategy, we first need to address the mechanisms responsible for maintaining genome stability. Importantly, given that most cancers ultimately develop genomic instability [9,10], such a prevention strategy might be applicable to many cancers.

### 2.7. Mutations in Cancer-Suppressor Genes

Colorectal cancers can develop as a result of three mutations, namely, mutations in adenomatous polyposis coli (APC), the ARF/p53 module, and KRAS [62]. Although it was previously thought that all of these mutations could be induced by errors during canonical replication, more recent evidence suggests that the situation might be more complicated. First, unlike mutations in TP53, loss of ARF function is usually caused by a large deletion in the CDKN2A locus [25,63,64,65], which is usually induced by genomic destabilization rather than simple replication error. Second, unlike mutations in the oncogene KRAS, loss of function of the tumor-suppressor genes APC, CDKN2A, and TP53 usually results from mutation of both allelic loci of the corresponding genes [66,67,68]. Finally, cells in the precancerous and benign stages that still show normal ARF/p53 function often develop with tetraploidy [69], and for their transformation to malignant cancers, TP53 (or CDKN2A) must be mutated in conjunction with mutations in four TP53 gene loci (or CDKN2A). Based on cancer frequencies, the mutation of four loci by random error is theoretically impossible. Indeed, if the probability of mutation in TP53 due to random polymerase error is 1 in 106 cells, then the probability of mutation in four copies in a tetraploid cell is 1 in 1024 cells (i.e., vanishingly unlikely). Therefore, based on the probabilities, it is theoretically impossible to induce all of the necessary tumor-suppressor mutations exclusively via random errors during normal replication.

How, then, are mutations that cause loss of function of tumor-suppressor genes induced? They are probably associated with genomic destabilization, including the induction of loss of heterozygosity (LOH) that often arises in association with genomic destabilization [23,25]. In this case, after one mutation is induced, mutations in other allelic gene loci can be induced by LOH in a “copy and paste” manner, mediated by erroneous HR between those loci [70,71]. During this process, the probabilities of mutation propagation or elimination are as high as 50%. In fact, LOH is often induced in cancer cells and is widely associated with the loss of function of tumor-suppressor genes [23]. Importantly, genomic destabilization is associated with the loss of function of tumor-suppressor genes and the expansion of cells with abrogated defense systems. In support of this conjecture, many cancers inevitably develop genomic instability.

## 3. Clonal Evolution of Cells with Abrogated Defense Systems

Each stage of cancer development involves the clonal evolution of cells with abrogated defense systems. Based on recent knowledge, multiple effects contribute to this evolution.

### 3.1. Genomic-Destabilization-Triggered Clonal Evolution

The aforementioned genomic destabilization is probably a major cause of the clonal evolution of cells with abrogated defense systems and the associated mutations in cancer-driver genes [7]. This issue is clearly illustrated by a recent study using a MEF model. Like many other normal cells in vivo and in vitro, MEFs usually undergo growth arrest with reduced levels of H2AX, which mediates active growth as well as DSB repair [50]. Therefore, such MEFs can remain quiescent when genome stability is maintained, but develop genomic instability under continuous growth stimulation due to the accumulation of replication-stress-associated DSBs [72,73]. Such genomic destabilization is associated with mutation induction, in which immortalized MEFs with mutations in the ARF/p53 module form a colony and H2AX expression is restored. Those immortalized MEFs eventually become predominant because they have resumed growth. These phenomena illustrate the clonal evolution process of cells mutated in the ARF/p53 module, which is triggered by genomic-destabilization-associated mutagenesis.

Clonal evolution associated with genomic destabilization is also observed when cancer cells acquire resistance to the anti-cancer drug camptothecin (CPT), which causes replication-stress-associated DSBs [7]. After treatment with CPT, most cancer cells undergo apoptosis due to the accumulation of DSBs. However, genomic destabilization is induced in cells that survive, eventually leading to the clonal evolution of cells with elevated resistance to CPT [7]. These results support the idea that the clonal evolution of cells resistant to drugs that cause replication-stress-associated DSBs is induced through genomic destabilization.

Together, these observations imply that replication-stress-associated genomic destabilization underlies the stepwise progression of cancer development, at least in regard to the clonal evolution of cells that have lost ARF/p53-dependent defense systems and have acquired resistance to the anti-cancer drug CPT [7]. This situation is conceptually similar to stress-induced mutagenesis [74,75,76,77]. Importantly, unlike errors randomly induced during canonical replication, replication-stress-triggered mutagenesis is tightly associated with the induction of mutations in cancer-driver genes, and therefore leads to the clonal evolution of cells with abrogated defense systems.

### 3.2. Transcription Variety and Epigenetic Alteration for Clonal Evolution

Unlike resistance to anti-cancer drugs that cause replication-stress-associated DSBs, resistance to drugs that inhibit growth-stimulation modules is often acquired without mutations that mediate resistance, as in the case of cancer cells resistant to vemurafenib [78]. Recently, the underlying mechanism of such resistance has been clarified by studies revealing the contribution of alterations in epigenetic regulation [78,79,80]. Cancers often develop by activating specific growth-acceleration modules [22,81,82]. Therefore, cancer cell growth could be specifically blocked by inhibitors that induce cancer cell death. However, because such states are achieved by specific growth-module activation via epigenetic regulation, they could be altered by changes in epigenetic status. In fact, under vemurafenib treatment, cells that show different growth-module dependence are clonally expanded through possible epigenetic fluctuation [78]. Thus, transcriptional variety and/or epigenetic alterations in cancer cells are also pathways that lead to the clonal evolution of cells resistant to certain types of anti-cancer drugs, and these pathways are probably distinct from the one mediated by genomic destabilization.

Unlike aging-associated cancers, infantile tumors often develop with very specific chromosomal translocations but without typical genomic instabilities, unlike many aging-associated cancers [83,84,85]. These tumors usually exhibit broad alteration in epigenetic regulation status [86,87]. Although it remains unclear what leads to infantile tumor evolution, epigenetic regulation alteration leading to the clonal expansion of cells is probably involved [88], as described previously for the development of resistance to vemurafenib [78]. This type of clonal evolution might constitute an alternative pathway for tumor growth, distinct from that of genomic-destabilization-triggered clonal evolution, which is usually caused by replication stress.

### 3.3. Association of Immune Response with Clonal Evolution

During tumor development in vivo, clonal evolution must be induced in association with adaptive responses to immune checkpoints, which regulate the immune system to prevent indiscriminate attack [89]. Although it remains unclear how the immune checkpoint is regulated, a recent study of the awakening of dormant cancer cells revealed the involvement of neutrophil extracellular traps (NETs), which are produced during inflammation [90]. In addition, clonal evolution during metastatic growth could occur in multiple contexts, in which inflammation is tightly associated [91,92,93,94]. Based on these findings, such clonal evolution in vivo probably includes inflammation-associated adaptive responses to the immune system, in addition to the development of transformed cells via genomic instability and/or epigenetic alteration. Importantly, genomic destabilization—especially in the case of CIN—is often associated with inflammation through the induction of cytosolic DNA and the associated activation of the cGAS/STING pathway [95,96,97,98]. Therefore, the genomic destabilization that induces the clonal evolution of cells undergoing cancer progression might be simultaneously associated with immune-checkpoint adaptation in vivo.

## 4. Perspectives

Based on accumulated evidence, it is now very likely that replication-stress-induced genomic destabilization is a major cause of mutations in cancer-driver genes, and that this leads to the clonal evolution of cells with abrogated defense systems. Mutation rates under replication stress are much higher than during canonical replication [7]. An attractive hypothesis raised by these findings is that it might be possible to reduce the rate of genomic-destabilization-associated mutations by maintaining genomic stability and thereby prevent cancer. Given that genomic destabilization is triggered by the accumulation of DSBs following replication stress, genomic stability could be increased by activating the DSB repair mechanisms that become defective in the repair of the replication-stress-associated DSBs during cancerogenesis.

## Figures and Tables

**Table 1:** Risks of mutation induction and genomic destabilization during normal replication and under replication stress.

	During Normal Replication	Under Replication Stress
MMR-proficient cells		
Operating DNA polymerases	Polα, Polδ, and Polε	TLS pols (*1)
Mutation rate	Low	High
Genomic destabilization risk and genomic instability type caused	No	CIN
Genomic-instability-associated alterations risking cancer-driver mutations	No	Gene amplification, LOH, and deletion (*2)
MMR-deficient cells		
Operating DNA polymerases	Polδ and Polε	TLS pols (*1)
Mutation rate	High	Very high
Genomic destabilization risk and genomic instability type caused	No	MSI
Genomic-instability-associated alterations risking cancer-driver mutations	No	LOH and deletion (*3)

*1: During the repair of replication stress-associated double strand breaks (DSBs), normal replicative polymerases δ and ε are usually inoperative; instead, low-fidelity translesion synthesis (TLS) polymerases are widely expressed, resulting in a mutagenic background, especially in MMR-deficient cells [7,17,18,19,20,21]. *2: In addition to point mutations, cancer-driver mutations are often associated with chromosomal instability (CIN), including amplification of oncogenes such as c-*Myc* and loss of heterozygosity (LOH), and deletions of tumor-suppressor genes [7,22,23,24]. *3: Although CIN is generally suppressed during microsatellite instability (MSI) induction, LOH and deletions causing loss of function of tumor-suppressor genes are often observed even in MSI-positive cancer cells, which drives their development [7,23,25].
